# Melatonin-induced *ZjWRKY11* and *ZjWRKY7* regulate triterpenoid biosynthesis in jujube (*Ziziphus jujuba* Mill.)

**DOI:** 10.3389/fpls.2026.1868423

**Published:** 2026-07-28

**Authors:** Cuiping Wen, Zhongtang Wang, Yuxiu Liu, Yuhan Wang, Chao Wang, Xingang Li

**Affiliations:** 1 College of Life Science, Dezhou University, Dezhou, China; 2 College of Forestry, Northwest Agriculture and Forestry University, Yangling, China; 3 Shandong Institute of Pomology, Taian, China; 4 College of Horticulture and Forestry, Tarim University, Alar, China

**Keywords:** jujube (*Ziziphus jujuba* Mill.), melatonin (MT), transcriptional regulation, triterpenoid metabolism, WRKY transcription factors

## Abstract

**Introduction:**

Triterpenoids are key bioactive metabolites in jujube (*Ziziphus jujuba* Mill.), yet their regulatory mechanisms remain unclear.

**Methods:**

In this study, jujube samples were treated with exogenous melatonin (MT), and triterpenoid accumulation was measured. Transcriptomic analysis was performed to identify differentially expressed genes in response to MT treatment. Key biosynthetic genes and transcription factors were further validated through overexpression and silencing assays, and promoter binding was examined to elucidate the regulatory mechanism.

**Results:**

Exogenous MT markedly enhanced the accumulation of major pentacyclic triterpenoids, with MT accumulation preceding triterpenoid biosynthesis. Transcriptomic analysis revealed that differentially expressed genes were significantly enriched in secondary metabolic pathways. Integrated analysis identified key biosynthetic genes (*ZjHMGR* and *ZjOSC1*) and MT-responsive WRKY transcription factors, particularly ZjWRKY11 and *ZjWRKY7*, that were strongly associated with triterpenoid accumulation. Functional assays demonstrated that overexpression of *ZjWRKY11* and *ZjWRKY7* markedly enhanced triterpenoid accumulation, whereas their silencing resulted in a significant reduction. Mechanistically, both transcription factors directly activated *ZjHMGR* and *ZjOSC1* by binding to their promoters, with *ZjWRKY11* showing stronger activation.

**Discussion:**

Collectively, our findings suggest that MT induces the expression of *ZjWRKY11* and *ZjWRKY7*, which positively regulate triterpenoid biosynthesis and may contribute to MT-associated triterpenoid accumulation through the activation of key biosynthetic genes. These findings provide insights into the transcriptional regulation of secondary metabolism and identify potential targets for future metabolic engineering efforts.

## 1 Introduction

Jujube (*Ziziphus jujuba* Mill.), a perennial woody fruit tree belonging to the Rhamnaceae family, has been cultivated for more than 7, 000 years and is widely valued for both fresh consumption and dried products (Li, 2015). In addition to its nutritional value, jujube has long been used in traditional medicine and is rich in diverse primary and secondary metabolites, including polysaccharides, amino acids, flavonoids, triterpenoids, and alkaloids (Liu et al., 2020; Zhang et al., 2023a). Among these compounds, pentacyclic triterpenoids are an important class of active secondary metabolites in jujube. Leaves, buds, and fruits are rich sources of these compounds, which possess a range of pharmacological effects, including anti-inflammatory, anti-tumor, sedative-hypnotic, and antioxidant activities (Wen et al., 2023a). Additionally, triterpenoids also serve as important defense metabolites in plants and play a crucial role in responding to both biotic and abiotic stressors (Deng et al., 2025; Wen et al., 2023b). In most plants, triterpenoid biosynthesis is initiated via the mevalonate (MVA) pathway, where key enzymes such as 3-hydroxy-3-methylglutaryl coenzyme A reductase (HMGR), farnesyl diphosphate synthase (FPS), and squalene synthase (SQS) catalyze the formation of squalene. Subsequently, squalene epoxidase (SQE) and oxidosqualene cyclases (OSCs) generate triterpene skeletons, which are further modified by cytochrome P450 monooxygenases (CYP450s) and various transferases to produce structurally diverse triterpenoids (Wen et al., 2022; Dong and Qi, 2025). The genes involved in the triterpenoid biosynthesis pathway and the regulatory transcription factors have been extensively studied in platycodon grandiflorus, soapberry, and various medicinal plants (Yao et al., 2020; Yu et al., 2021; Zheng et al., 2024). However, the biosynthetic metabolic mechanism of triterpenoids is complex. In recent years, the active substances in various tissues and fruits of jujube have been identified and quantitatively analyzed (Pan et al., 2025). In our previous research, multiple key structural genes for jujube triterpenoid biosynthesis were also identified through transcriptome and metabolome analyses (Wen et al., 2023a; Zhang et al., 2022). However, the research on the complex regulatory mechanism of its biosynthesis is relatively scarce. In particular, the accumulation and synthesis mechanism of triterpenoids under specific hormone induction is still unclear, which to some extent hinders the improvement of jujube fruit quality and the progress of resistance breeding.

MT is a highly conserved compound that is considered a potential growth stimulant in plants. It has attracted much attention due to its multiple regulatory roles in plant development and response regulation (Ahammed et al., 2020). As a direct antioxidant and a multifunctional regulator of stress-responsive genes encoding antioxidant enzymes, MT plays a key role in mitigating damage induced by abiotic stresses such as drought, high temperature, and salinity (Chen et al., 2020; Zhang et al., 2015). Notably, the identification of a putative MT receptor, CAND2/PMTR1, in Arabidopsis thaliana has offered new perspectives on MT-mediated signaling pathways (Wei et al., 2018). MT treatment has been shown to enhance the profiles of various plant hormones involved in secondary metabolism and growth development. Growing evidence suggests that melatonin (MT) promotes the accumulation of secondary metabolites by modulating hormone balance and transcriptional reprogramming (Dou et al., 2022; Ma et al., 2021; Yu et al., 2023). For instance, exogenous MT enhances phenolic accumulation in rosemary callus, regulates cuticular wax biosynthesis in blueberry (Li et al., 2024), and stimulates saponin biosynthesis in medicinal plants through complex hormonal and transcriptional networks. Despite these advances, the transcriptional regulators that mediate MT-induced secondary metabolism remain poorly understood in many plant species, including jujube. Specifically, the role of MT in regulating triterpenoid biosynthesis in jujube and its underlying molecular mechanisms remain largely unexplored. Therefore, identifying melatonin-responsive transcription factors is essential for elucidating the molecular mechanisms underlying MT-regulated triterpenoid biosynthesis in jujube.

Transcription factors are key mediators that translate upstream signaling cues into specific transcriptional responses. Among the various transcription factor families, WRKY proteins are particularly important because of their roles in hormone signaling, stress responses, and secondary metabolite biosynthesis (He et al., 2025). Many transcription factors, including WRKY, NAC, and AP2/ERF, are critically involved in the transcriptional regulation of MT, and they may modulate the expression of downstream genes across multiple biological processes (Han et al., 2021). Therefore, identifying melatonin-responsive transcription factors is essential for understanding the molecular mechanisms underlying MT-regulated secondary metabolism. Among the numerous transcription factor families, the WRKY family is a highly conserved and functionally diverse group in higher plants, widely involved in various biological processes such as plant growth and development, stress response, and secondary metabolism regulation. The typical feature of WRKY transcription factors is that their DNA binding domain contains a highly conserved WRKYGQK sequence and a zinc finger motif, which can specifically recognize and bind to the W-box (C/T)TGAC(C/T) cis-element in the promoter of target genes (Brand et al., 2013). Since the WRKY transcription factor *SPF1* was first identified in *Ipomoea batatas* (Ishiguro and Nakamura, 1994), this family has been systematically identified in various plants such as *Arabidopsis*, *Oryza sativa*, and *Panax ginseng* (Di et al., 2021; Abdullah-Zawawi et al., 2021), and it has been confirmed to play a key role in growth and development, responses to biological and abiotic stresses, as well as regulation of secondary metabolism (Agarwal et al., 2011; Khoso et al., 2022). Increasing evidence indicates that WRKY transcription factors directly regulate terpenoid biosynthesis in medicinal plants. For example, *AaWRKY1* positively regulates artemisinin biosynthesis in *Artemisia annua* (Ma et al., 2009), whereas *PgWRKY21* and *PgWRKY101* participate in ginsenoside biosynthesis in *Panax ginseng* (Wang et al., 2025). In addition, overexpression of *PnWRKY35* activates key triterpenoid biosynthetic genes and promotes triterpenoid accumulation in *Panax notoginseng* (Li et al., 2025a). These findings indicate that WRKY transcription factors frequently act as molecular links between upstream signaling pathways and downstream terpenoid biosynthetic genes. Therefore, melatonin-responsive WRKY transcription factors may represent important regulators of triterpenoid biosynthesis in jujube.

Although the functions of WRKY transcription factors have been deeply studied in various plants, related research on jujube is still relatively limited Chen et al. (2019) were the first to identify WRKY transcription factors in ‘Junzao’ and ‘Winter Jujube’ at the genomic level, obtaining 61 and 52 *ZjWRKY* members respectively. Through transcriptome analysis, further identified WRKY genes in jujube that respond to drought and salt stress, and revealed their evolutionary and expression patterns (Chen et al., 2019). Accumulating evidence indicates that WRKY transcription factors serve as key mediators in the regulation of secondary metabolite biosynthesis induced by methyl jasmonate (Wen et al., 2023b). The combined analysis of metabolomics and transcriptomics also suggests that WRKY may be involved in the phenylpropanoid metabolism and fructose metabolism pathways in jujube (Yuan et al., 2025). Although melatonin has been reported to regulate secondary metabolism and WRKY transcription factors have been implicated in terpenoid biosynthesis, whether melatonin-responsive WRKY transcription factors participate in triterpenoid biosynthesis in jujube remains unknown. Therefore, elucidating the relationship between MT signaling, WRKY transcription factors, and triterpenoid biosynthesis represents an important research objective.

In this study, we hypothesized that MT-induced triterpenoid accumulation may be associated with the activation of specific WRKY transcription factors. To test this hypothesis, we identified two candidate genes, *ZjWRKY11* and *ZjWRKY7*, and investigated their roles in MT-induced triterpenoid accumulation. By integrating physiological, transcriptomic, and molecular approaches, we aimed to elucidate the regulatory network underlying MT-mediated triterpenoid biosynthesis in jujube. This study not only provides new insights into the molecular mechanisms of secondary metabolism regulation but also offers potential strategies for improving fruit quality and stress resistance in jujube.

## 2 Materials and methods

### 2.1 Plant materials

Wild jujube seeds were sourced from the experimental station in Qingjian, Yulin, China. All seedlings were maintained in climate-controlled greenhouse at 24 °C with 16/8h (ligh/dark) photoperiod. Three-week-old seedlings demonstrating homogeneous growth were selected for experimental use. For MT treatment, MT (Sigma-Aldrich, St. USA) was dissolved in a small volume of absolute ethanol to generate a concentrated stock solution and then diluted with distilled water to obtain final concentrations of 50, 100, and 200 μM. The final ethanol concentration was maintained below 0.1% (v/v) in all solutions. Given the photosensitive nature of MT, stock and working solutions were prepared under dim-light conditions and stored in amber bottles to minimize light-induced degradation. Fresh working solutions were prepared immediately before each application. Uniform three-week-old seedlings were sprayed with MT solution until runoff, three times daily (08:00, 14:00, and 20:00). Control plants were treated with the corresponding solvent solution lacking MT. All treatments were conducted under the same controlled growth conditions. Leaves were harvested at 0, 24, 72, 120, 168, and 216 h following the first application, rapidly frozen in liquid nitrogen, and stored at −80 °C prior to metabolite and gene expression analyses. Tobacco plants were grown in a light incubator under a 16 h/8 h light (24 °C)/dark (18 °C) photoperiod. “NC89” tobacco tissue culture seedlings were cultured in a sterile culture room.

### 2.2 Extraction and content determination of triterpenoids

The total triterpene content (TTC) was determined using the vanillin-glacial acetic acid method described by Wei et al.34 Approximately 0.5 g of powdered freeze-dried samples were ground and dissolved in 5 mL of 90% methanol/H_2_O (v/v). Plant extract (20 μL) was combined with acidic vanillin reagent (150 μL). Subsequently, 500 μL of perchloric acid and 2.25 mL of glacial acetic acid were added to the mixture. The extraction and HPLC-based quantification of pentacyclic triterpenoids were conducted in accordance with the method described by Wen et al (Wen et al., 2023a).

### 2.3 Determination of melatonin content

Determination of MT levels was performed using LC-MS/MS based on a modified method described by Chmur et al. (Chmur and Bajguz, 2023). Separation was achieved on a C18 column (2.1 × 100 mm, 1.8 μm) at 40 °C. The mobile phase consisted of water (containing 0.1% formic acid) and methanol at a flow rate of 0.3 mL/min.

### 2.4 RNA extraction, qRT-PCR and transcriptome analysis

Following the manufacturer’s protocol, total RNA was isolated from plant samples with the Plant Polysaccharide & Polyphenol RNA Kit (FOREGENE, Chengdu, China). The quality of the extracted RNA was verified by agarose gel electrophoresis, while concentrations were determined using a NanoDrop 2000 spectrophotometer (Thermo Fisher Scientific, USA). Reverse transcription to generate first-strand cDNA was conducted using the FOREGENE Master Premix RT Easy™ II Kit. For amplification, qRT-PCR was performed with Real Time PCR Easy™-SYBR Green I reagents on a CFX96 Touch Real-Time PCR Detection System (Bio-Rad, USA). The primers are shown in **Supplementary Table 1**. For transcriptome analysis, leaves from control and 100 μM MT-treated seedlings were collected after 168 h of treatment, with three biological replicates per treatment. RNA libraries were sequenced on the DNBSEQ T7 platform. Clean reads were aligned to the Chinese jujube reference genome (NCBI accession: GCA_00183579.2) using HISAT2, and gene expression levels were calculated as FPKM values. Differentially expressed genes were identified using DESeq2 (FDR < 0.05 and |log2FC| ≥ 1). Detailed sequencing information is provided in **Supplementary Table 5**. Previously characterized genes involved in jujube triterpenoid biosynthesis (Wen et al., 2022, Wen et al., 2023a), including *ZjHMGS, ZjHMGR, ZjSQE, ZjOSC1, ZjOSC2*, and *ZjCYP450*, were retrieved and their expression patterns under MT treatment were analyzed using the RNA-seq data generated in this study. Differentially expressed WRKY genes were extracted from the RNA-seq data, and candidate genes for further analyses were selected based on their expression patterns and homology to previously characterized terpenoid-related WRKY transcription factors. Raw RNA-seq data are available in the NCBI SRA under accession number PRJNA1466623. The RNA-seq dataset has been partially used in another accepted manuscript; however, the analyses and conclusions presented here are independent and have not been reported previously.

### 2.5 Vector construction and identification of transgenic plants

The key gene construct was generated by cloning a gene-specific fragment, amplified from jujube leaf cDNA, into the pTRV2 vector. For overexpression, the open reading frame of the target gene was inserted into the pC2300-green fluorescence protein (GFP) vector under the control of the CaMV 35S promoter. The primers are shown in **Supplementary Table 1**. *Agrobacterium tumefaciens* GV3101 harboring the overexpression or TRV-based silencing constructs was cultured to an OD600 of approximately 0.8 and resuspended in infiltration buffer. Bacterial suspensions were infiltrated into the abaxial side of fully expanded jujube leaves using a needleless syringe. Empty vectors were used as controls. Leaf samples were collected 5 d after infiltration for gene expression and triterpenoid analyses. Three independent biological replicates were performed for each treatment. Following the *Agrobacterium* leaf disk method, the overexpression vector was transformed into tobacco “NC89” and selected on 50 mg/mL kanamycin. More than ten independent kanamycin-resistant transformants were obtained for each construct. Transgene integration was confirmed by PCR and expression levels were evaluated by qRT-PCR. Three independent T0 transgenic plants were used for subsequent analyses. For metabolite analysis, fully expanded leaves were harvested from four-week-old transgenic tobacco plants grown under controlled environmental conditions.

### 2.6 Subcellular localization

The coding sequence (CDs) of the target gene was amplified and inserted into the pCambia2300-GFP vector using specific primers containing BamHI and SalI restriction sites (**Supplementary Table 1**). Both the resulting fusion construct and the empty vector were introduced into GV3101 competent cells for transient expression in *N. benthamiana* leaves. Fluorescence signals were visualized under a confocal laser-scanning microscope (TCS SP8 SR; Leica Zeiss, Germany).

### 2.7 Phylogenetic tree analysis

Phylogenetic analysis of the WRKY transcription factors involved in regulating flavonoid biosynthesis and responses to phytohormones such as MT was performed using MEGA v.6 with default settings and the neighbor-joining method (**Supplementary Table 2**). Branch support was evaluated using 1, 000 bootstrap replicates (accessed on 8 March 2026).

### 2.8 Glucuronidase and luciferase activity assays

Promoter regions of the genes were cloned into the pC0390GUS and pGreenII 0800-LUC reporter vectors, whereas the coding sequences of the target genes were inserted into the pGreen62-SK effector vector (**Supplementary Tables 1**, **S4**). Each construct was introduced into Agrobacterium tumefaciens strain GV3101 (Weidi, Shanghai) containing the pSoup helper plasmid. Bacterial suspensions carrying the reporter and effector constructs were combined and infiltrated into the abaxial surface of tobacco leaves. After 48–60 hours, leaf tissues were harvested for GUS histochemical staining and luciferase activity assays using a commercial kit (Beyotime, Nanjing).

### 2.9 Yeast one-hybrid assay

To construct the bait vectors, promoter fragments of *ZjHMGR* (223 bp) and *ZjOSC1* (254 bp) containing putative W-box cis-elements (TTGACC/T) were cloned into the pAbAi plasmid. The resulting pAbAi-*ZjHMGR* and pAbAi-*ZjOSC1* constructs were linearized and integrated into the genome of the Y1HGold yeast strain. Transformants were selected on SD/-Ura medium, and the optimal aureobasidin A (AbA) concentrations used to suppress self-activation were subsequently determined. Sequences encoding TFs were cloned into pGADT7 and subsequently introduced into Y1HGold cells already carrying the pAbAi-gene constructs. The empty vectors pGADT7-P53 and pGADT7 served as positive and negative controls, respectively.

### 2.10 Data analysis

Data visualization and statistical analyses were conducted using GraphPad Prism 9 (San Diego, CA, USA), Tbtools, and the OmicShare Tools platform (https://www.omicshare.com/tools/; accessed 20 March 2026). Data are presented as mean ± SE from at least three independent biological replicates. For comparisons between two groups, Student’s t-test was used. For experiments involving more than two groups, one-way ANOVA followed by Tukey’s multiple-comparison test was performed. Differences were considered statistically significant at p < 0.05.

## 3 Results

### 3.1 Exogenous MT induces the accumulation of triterpenoids and the production of MT in jujube

**Figure 1:**
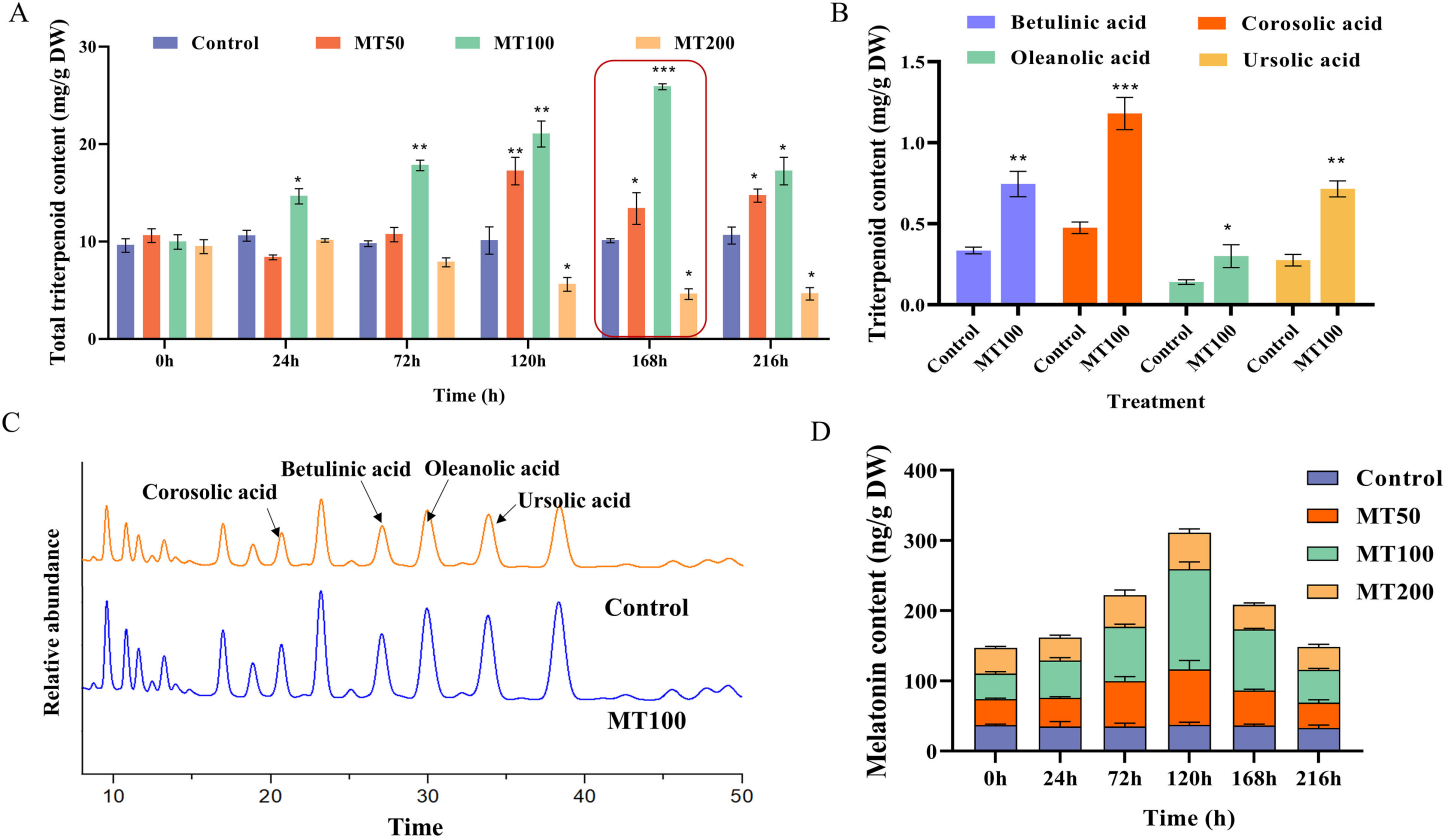
MT induces the accumulation of triterpenoids in jujube. **(A)** Changes in total triterpenoid content under different concentrations of MT treatment; **(B)** Changes in the content of individual pentacyclic triterpenoids induced by MT; **(C)** Liquid chromatogram of pentacyclic triterpenoids; **(D)** MT content. Data are presented as mean ± SE from three independent cultivations, each with three biological replicates. P < 0.05 versus the control (one-way ANOVA with Tukey’s test). *p < 0.05, **p < 0.01, ***p < 0.001.

To elucidate the regulatory role of MT in triterpenoid accumulation in jujube, seedlings were treated with different concentrations of MT (50, 100, and 200 μM), and young leaves were sampled at multiple time points to determine changes in triterpenoid content. Treatment with 100 μM MT led to a progressive increase in total triterpenoid levels, reaching a maximum at 168 h (25.9 mg/g DW), which was approximately 2.6-fold higher than that of the control. Although 50 μM MT also promoted triterpenoid accumulation, the effect was not statistically significant. In contrast, treatment with 200 μM MT resulted in a declining trend in triterpenoid content, suggesting an inhibitory effect on triterpenoid biosynthesis at higher concentrations (**Figure 1A**). To explore how 100 μM MT at 168 h affects major pentacyclic triterpenoids, four key compounds—corosolic acid, betulinic acid, oleanolic acid, and ursolic acid—were quantified using HPLC. MT treatment significantly elevated all four triterpenoids. In particular, betulinic acid, corosolic acid, and ursolic acid increased by roughly 2.3, 2.5, and 2.6 times, respectively (**Figures 1B, C**). These findings demonstrate that 100 μM MT treatment for 168 h markedly enhances triterpenoid accumulation in jujube leaves.

In addition, exogenous MT application altered MT levels in jujube leaves. The MT content increased significantly after treatment, reaching a peak of 135.1 ng/g DW at 120 h, whereas triterpenoid accumulation was delayed and showed a significant increase only at 168 h (**Figure 1D**). This temporal discrepancy indicates that MT accumulation precedes triterpenoid biosynthesis, implying a potential regulatory role of MT in activating the triterpenoid biosynthetic pathway. Furthermore, MT levels exhibited clear time- and concentration-dependent responses following exogenous MT application. Compared with the control, all MT-treated groups showed a gradual increase in MT content from 0 to 120 h. Notably, the MT100 group displayed the highest accumulation, peaking at 120 h and significantly exceeding the other treatments. After 120 h, MT levels declined progressively at 168 h and 216 h. Taken together with the observed triterpenoid accumulation patterns, these results further support that MT accumulation occurs prior to triterpenoid biosynthesis, highlighting its potential role as an upstream regulator of this metabolic process.

### 3.2 Transcriptome analysis identifies differential expression and secondary metabolic pathways

**Figure 2:**
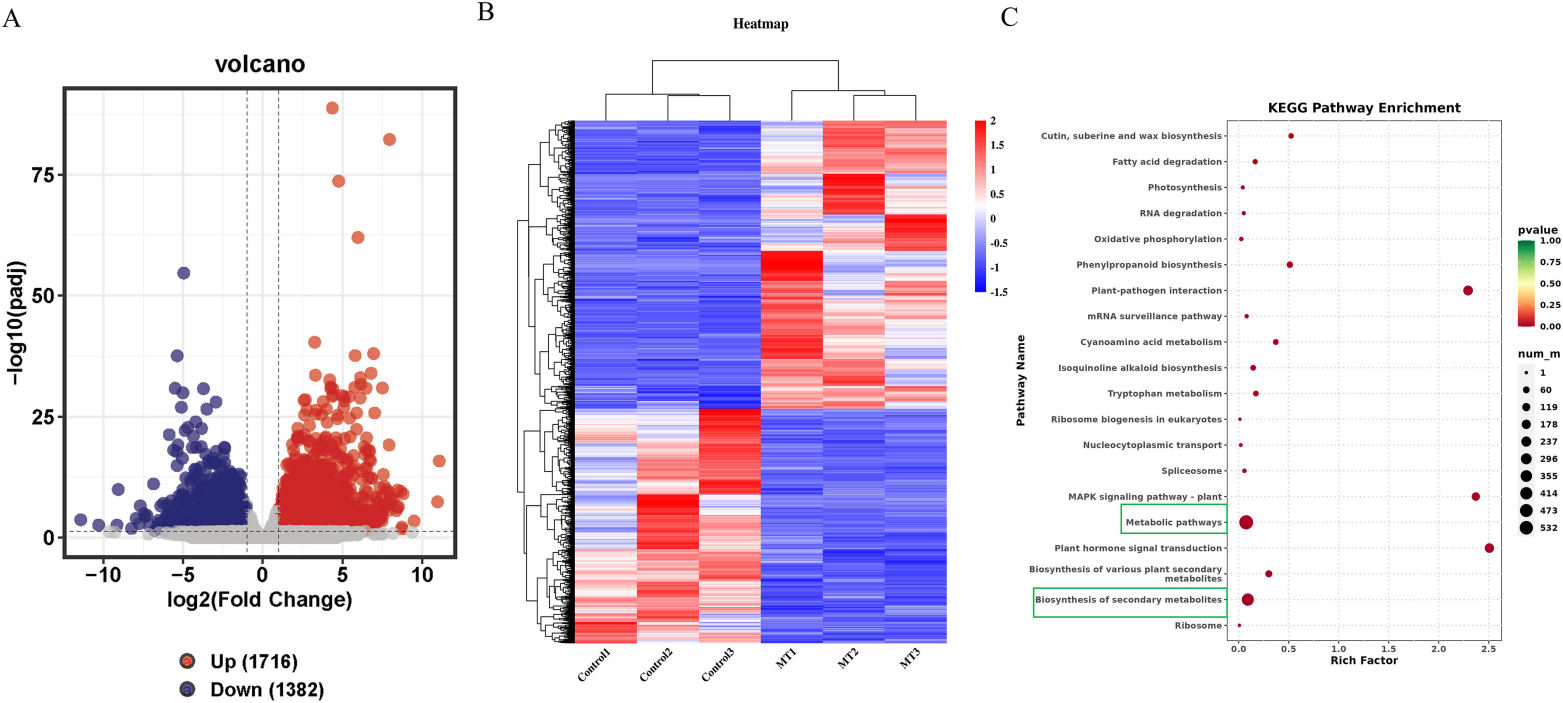
Transcriptome analysis. **(A)** Number of differentially expressed genes; **(B)** Expression levels of differentially expressed genes between MT treatment and control groups; **(C)** KEGG enrichment analysis of differentially expressed genes.

To further investigate the molecular mechanisms underlying MT-induced triterpenoid accumulation, transcriptome analysis was performed on jujube leaves treated with 100 μM MT for 168 h and corresponding control samples. A large number of differentially expressed genes (DEGs) were identified following MT treatment, including 1, 716 upregulated and 1, 382 downregulated genes (**Figure 2A**; **Supplementary Table 5**). A heatmap analysis further illustrated the global expression patterns of these DEGs, revealing pronounced transcriptional differences between MT-treated and control groups, with the majority of genes exhibiting significant expression changes upon treatment (**Figure 2B**; **Supplementary Table 6**). Furthermore, KEGG enrichment analysis revealed that these DEGs were mainly linked to metabolic pathways, especially those related to the biosynthesis of secondary metabolites (**Figure 2C**; **Supplementary Table 7**).

### 3.3 Expression of triterpenoid biosynthetic genes and identification of key transcription factors

**Figure 3:**
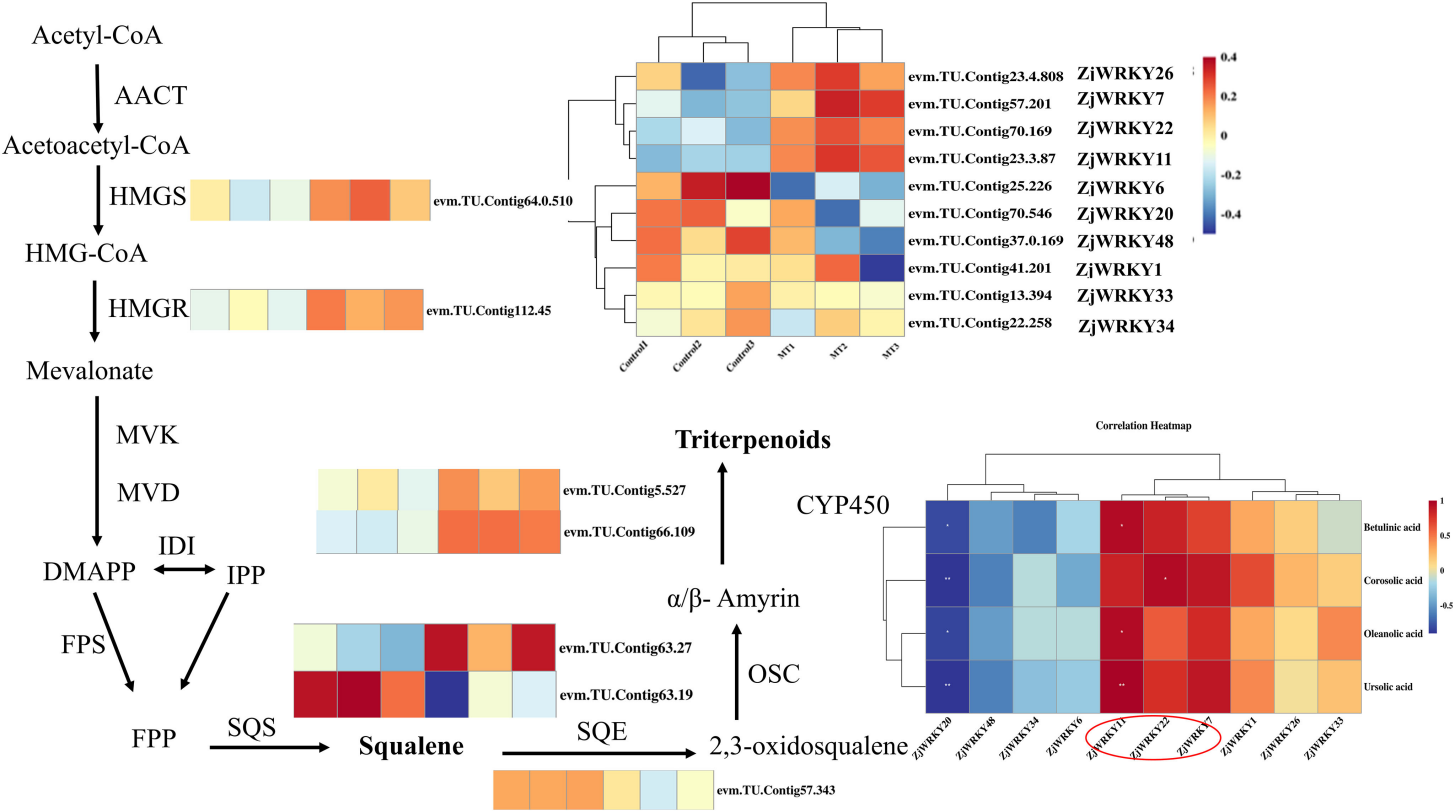
Gene expression of jujube triterpene biosynthesis pathway, expression of key WRKY transcription factors and correlation analysis. The symbols * and ** represent statistical significance levels ( p < 0.05, ** p < 0.01).

To elucidate the molecular basis of MT-induced triterpenoid biosynthesis in jujube, we analyzed the expression profiles of genes involved in both the mevalonate (MVA) and methylerythritol phosphate (MEP) pathways, as well as downstream triterpenoid biosynthetic genes, using RNA-seq data from MT-treated and control samples. The expression profiles of all identified genes, including MVA- and MEP-pathway genes and all OSC family members detected in the transcriptome dataset, are provided in **Supplementary Table 9**. Overall, MT treatment induced transcriptional changes in genes involved in isoprenoid biosynthesis, although the responses varied among individual genes. Genes exhibiting pronounced transcriptional responses were selected for heatmap visualization, which was generated using row-scaled expression values to highlight relative expression patterns. Among these, *ZjHMGS, ZjHMGR, ZjOSC1*, and *ZjCYP450* genes were markedly upregulated following MT treatment, whereas *ZjSQE, ZjOSC2*, and most other homologous genes displayed relatively weak or no significant transcriptional responses (**Figure 3**; **Supplementary Table 9**). Among all *OSC* family members, *ZjOSC1* was the only one showing substantial upregulation, whereas other *OSC* genes exhibited marginal or no significant expression changes under MT treatment (**Supplementary Table 9**). The expression profiles of genes showing low expression levels or limited responsiveness, including additional MVA- and MEP-pathway genes and other *OSC* family members, are presented in **Supplementary Table 9**. These findings suggest that MT induces coordinated transcriptional regulation of multiple genes associated with triterpenoid biosynthesis, with several key biosynthetic genes exhibiting stronger transcriptional responses under the experimental conditions used in this study.

To comprehensively evaluate transcriptional responses to MT, all differentially expressed transcription factors (TFs) were classified according to PlantTFDB annotation. Multiple TF families were identified, including WRKY, MYB, AP2/ERF, NAC, bHLH, bZIP, and C2H2 (**Supplementary Table 8**). The expression profiles of all MT-responsive WRKY genes are presented in **Supplementary Figure 1**. Among these families, WRKY genes exhibited relatively strong induction following MT treatment. Combined with our previous studies demonstrating the involvement of WRKY transcription factors in jujube triterpenoid biosynthesis, the WRKY family was prioritized for further analysis. Transcriptomic analysis revealed that WRKY transcription factors were responsive to MT treatment. Based on the expression profiles of MT-responsive WRKY genes and their sequence similarity to previously characterized terpenoid-related WRKY transcription factors from other plant species, ten candidate WRKY genes were selected for further functional analyses. Among them, *ZjWRKY26, ZjWRKY7, ZjWRKY11*, and *ZjWRKY22* showed increased expression upon MT induction. To further identify the key regulatory transcription factors, we conducted a transcriptional level analysis of 10 transcription factors after MT induction. We found that the expression levels of *ZjWRKY11*, *ZjWRKY22, ZjWRKY7* were significantly induced and elevated by MT 168 hours later, reaching the peak expression level. The expressions of other *ZjWRKY6, ZjWRKY20, ZjWRKY48, ZjWRKY34* showed varying degrees of downward trends at different times of MT treatment (**Supplementary Figure 2**). Furthermore, correlation analysis between triterpenoid content and transcription factor expression levels suggested that *ZjWRKY11*, *ZjWRKY22*, and *ZjWRKY7* are likely key regulators involved in MT-induced triterpenoid accumulation in jujube (**Figure 3**).

### 3.4 *ZjWRKY11* and *ZjWRKY7* regulate triterpenoid biosynthesis by modulating key structural genes

**Figure 4:**
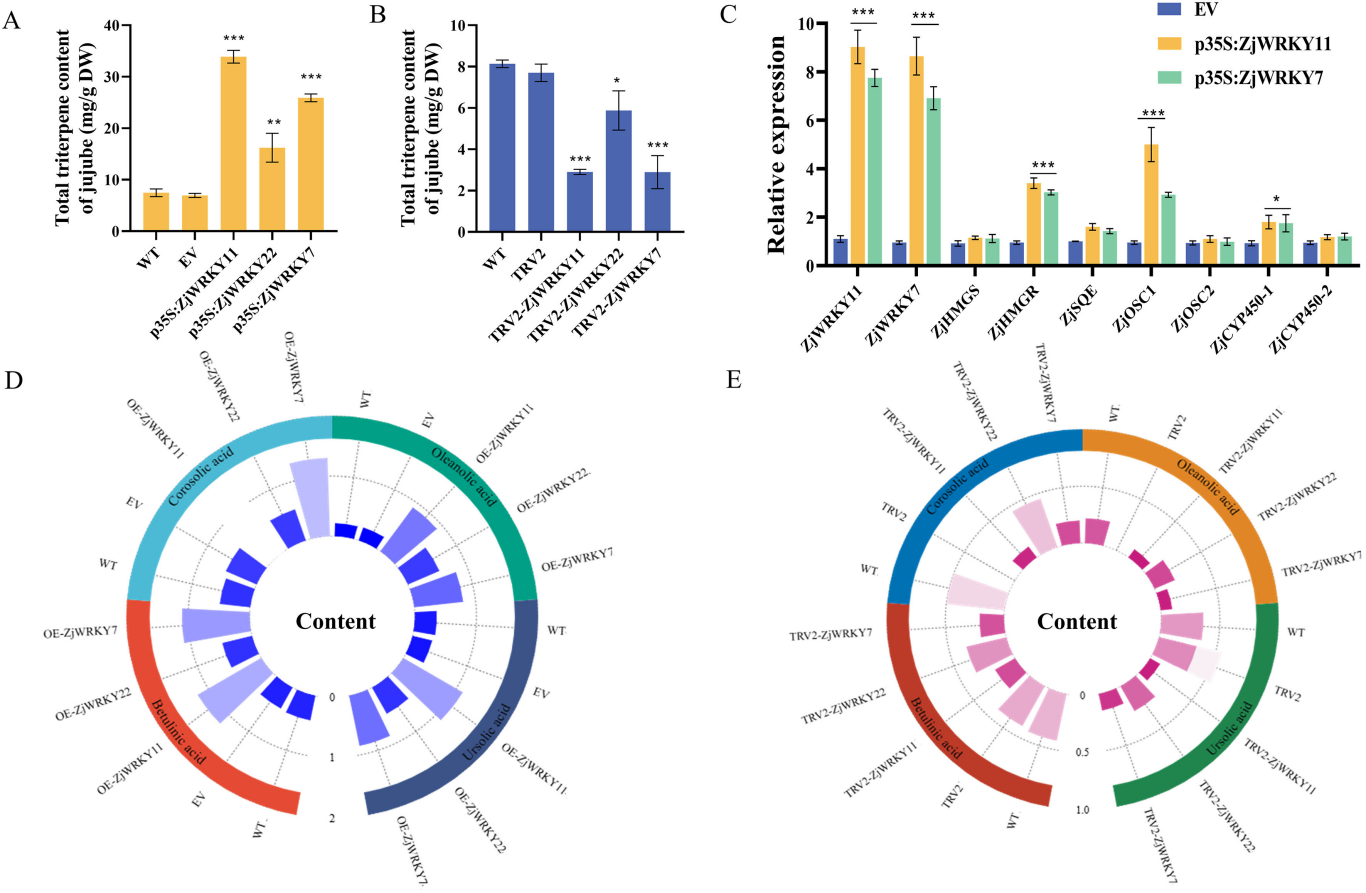
Functional identification of *ZjWRKY11* and *ZjWRKY7* transcription factors in the synthesis of jujube triterpenoids. **(A, B)** Effects of transient overexpression and transient silencing of key genes on total triterpenoid content; **(C)** Effects of overexpression of *ZjWRKY11* and *ZjWRKY7* on the expression of genes in the triterpenoid biosynthesis pathway; **(D, E)** Effects of transient overexpression and transient silencing of *ZjWRKY11* and *ZjWRKY7* transcription factors on the content of key monomeric pentacyclic triterpenoids in jujube. Data are means ± SE from three independent cultivations each with three biological replicates. Values marked with asterisks are significantly different from those of control plants (Student’s t-test; p < 0.05). *p < 0.05, **p < 0.01, ***p < 0.001.

To determine which of the MT-responsive WRKY transcription factors are functionally involved in triterpenoid biosynthesis, transient overexpression and gene-silencing assays were performed for *ZjWRKY11, ZjWRKY22*, and *ZjWRKY7*. Overexpression of *ZjWRKY11* and *ZjWRKY7* markedly increased total triterpenoid content, reaching approximately 3–4-fold higher levels than those in wild-type plants, whereas silencing of either gene reduced triterpenoid accumulation by 2–3 fold (**Figures 4A, B**). In contrast, manipulation of *ZjWRKY22* expression had no significant effect on triterpenoid content. These results indicate that *ZjWRKY11* and *ZjWRKY7*, but not *ZjWRKY22*, play positive regulatory roles in triterpenoid biosynthesis and were therefore selected for subsequent mechanistic analyses. Consistently, the levels of major pentacyclic triterpenoids—corosolic acid, oleanolic acid, betulinic acid, and ursolic acid—were significantly elevated upon overexpression and decreased upon silencing of *ZjWRKY11* and *ZjWRKY7* (**Figures 4D, E**), indicating that these two transcription factors are key regulators of MT-induced triterpenoid biosynthesis. Mechanistically, overexpression of *ZjWRKY11* and *ZjWRKY7* markedly enhanced the transcription of *ZjHMGR* and *ZjOSC1*, while genes such as *ZjHMGS, ZjSQE*, *ZjOSC2*, and *ZjCYP450–2* were largely unaffected (**Figure 4C**). Conversely, silencing of *ZjWRKY11* and *ZjWRKY7* significantly suppressed the expression of *ZjHMGR* and *ZjOSC1* (**Supplementary Figure 3**), suggesting that these transcription factors specifically regulate key enzymatic steps in the triterpenoid biosynthetic pathway.

**Figure 5:**
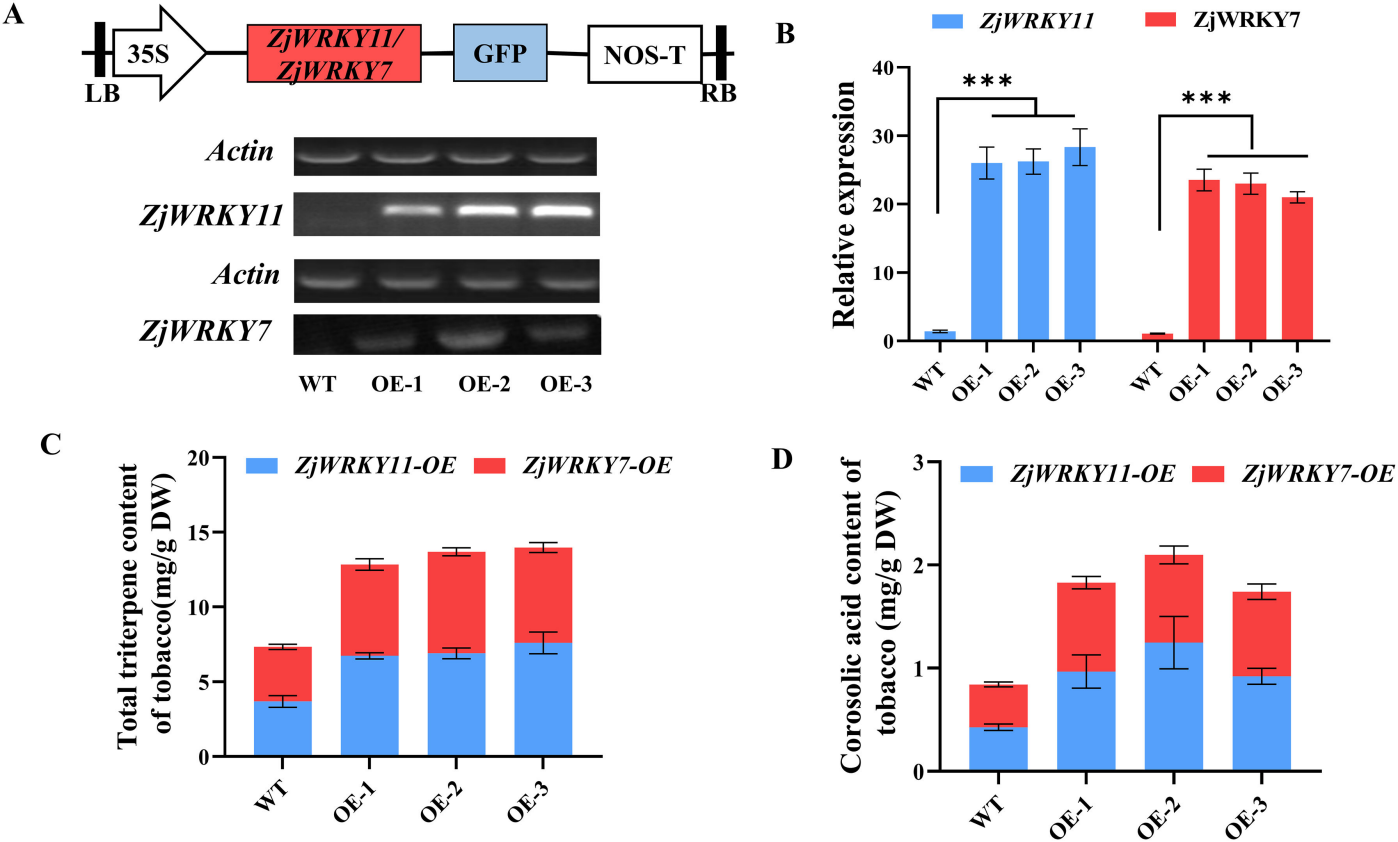
Effects of *ZjWRKY11* and *ZjWRKY7* overexpression on the total triterpenoid and corosolic acid content in transgenic tobacco and WT. **(A, B)** *ZjWRKY11* and *ZjWRKY7* expression levels in the overexpression lines (OE-1, OE-2, and OE-3) were measured using RT-PCR and qRT-PCR. **(C, D)** Effects of *ZjWRKY11* and *ZjWRKY7* overexpression on the total triterpenoid and corosolic acid content in transgenic tobacco and WT. The bars indicate mean ± SE. Values marked with asterisks are significantly different from those of control plants (Student’s t-test; p < 0.05). ***p < 0.001.

To further validate their regulatory roles, *ZjWRKY11* and *ZjWRKY7* were heterologously expressed in *Nicotiana tabacum* (cv. NC89), where semi-quantitative PCR and qRT-PCR confirmed strong transgene expression (**Figures 5A, B**; **Supplementary Figure 5**). Correspondingly, transgenic plants exhibited significantly increased levels of total triterpenoids and corosolic acid compared with controls, with total triterpenoid content rising by approximately 1.9-fold (*ZjWRKY11*) and 1.8-fold (*ZjWRKY7*), and corosolic acid increasing by about 2.7-fold and 2.0-fold, respectively (**Figures 5C, D**). Three independent transgenic lines (OE-1, OE-2, and OE-3) were analyzed for each construct. All lines exhibited elevated triterpenoid accumulation relative to WT plants, although the magnitude of increase varied slightly among lines, indicating that the positive effect of *ZjWRKY11* and *ZjWRKY7* on triterpenoid biosynthesis was reproducible across independent transformation events. *ZjWRKY11* and *ZjWRKY7* may be involved in the biosynthesis of triterpenoids induced by MT by regulating the expression of key triterpenoid synthesis genes *ZjHMGR* and *ZjOSC1*.

To determine the roles of *ZjHMGR* and *ZjOSC1* in triterpenoid biosynthesis, transient overexpression and gene silencing assays were performed. Transient overexpression of *ZjHMGR* or *ZjOSC1* significantly increased triterpenoid accumulation in jujube, whereas silencing of these genes resulted in a marked reduction in triterpenoid content (**Supplementary Figure 4A**). Furthermore, *ZjHMGR* and *ZjOSC1* were stably transformed into *Nicotiana tabacum*, and independent transgenic lines were obtained. Measurement of total triterpenoid content revealed that transgenic plants exhibited a 2–3-fold increase compared with wild-type controls (**Supplementary Figures 4B, C**). These results indicate that *ZjHMGR* and *ZjOSC1* play essential roles in triterpenoid biosynthesis, providing a foundation for further investigation of transcriptional regulatory mechanisms controlling triterpenoid production.

### 3.5 Phylogenetic analysis and subcellular localization of *ZjWRKY11* and *ZjWRKY7*

**Figure 6:**
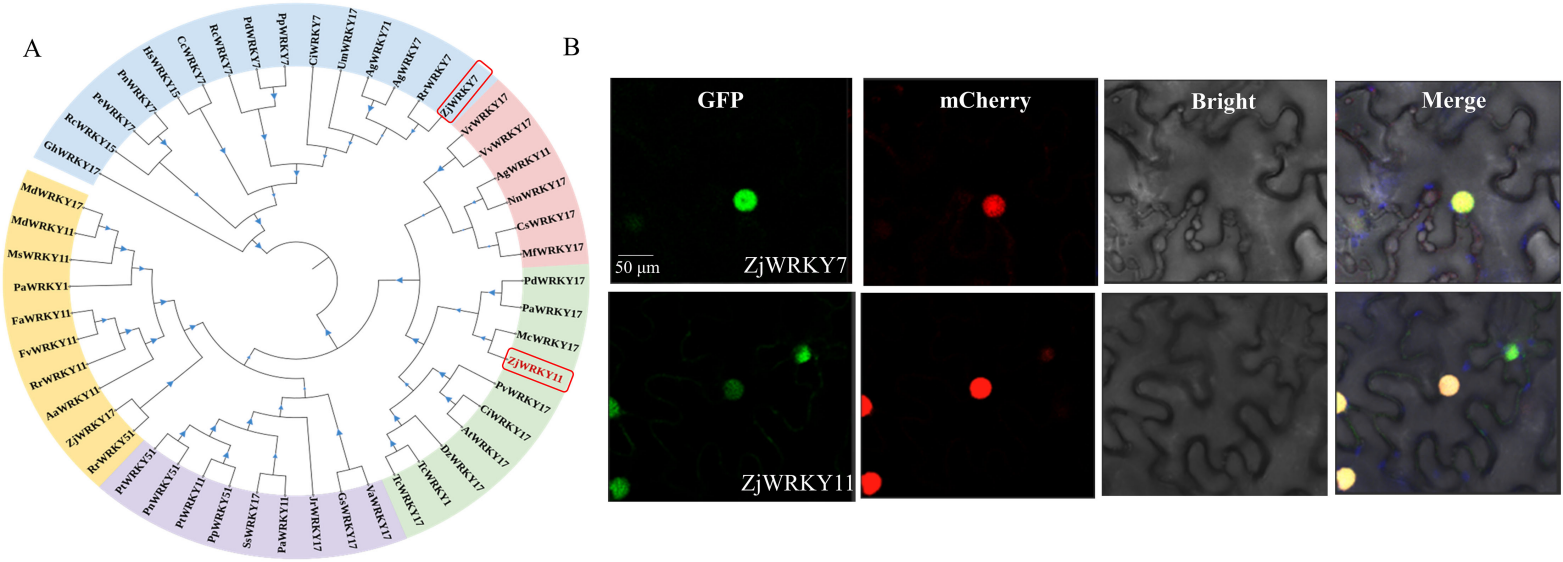
Phylogenetic and subcellular localization analysis of the ZjWRKY11 and ZjWRKY7 systems. **(A)** Phylogenetic tree; **(B)** Subcellular localization. The experiments were repeated three times. (Scale bars = 50 μm).

To characterize the functional properties of *ZjWRKY11* and *ZjWRKY7*, their protein sequences were subjected to phylogenetic analysis together with representative WRKY transcription factors from other plant species. The results showed that ZjWRKY11 clustered most closely with McWRKY17, PaWRKY17, and PdWRKY17, while ZjWRKY7 exhibited the highest sequence similarity to RrWRKY7 and AgWRKY7 (**Figure 6A**), suggesting potential functional conservation among these homologs. In addition, subcellular localization assays were performed to determine the intracellular distribution of these proteins. Fusion constructs (*ZjWRKY11*-GFP and *ZjWRKY7*-GFP) were transiently expressed in transgenic tobacco leaves carrying a nuclear marker. Fluorescence signals from both fusion proteins were exclusively detected in the nucleus (**Figure 6B**), consistent with their predicted roles as transcription factors regulating gene expression.

### 3.6 *ZjWRKY11* and *ZjWRKY7* activate the promoters of *ZjHMGR* and *ZjOSC1* and promote triterpenoid biosynthesis in jujube

**Figure 7:**
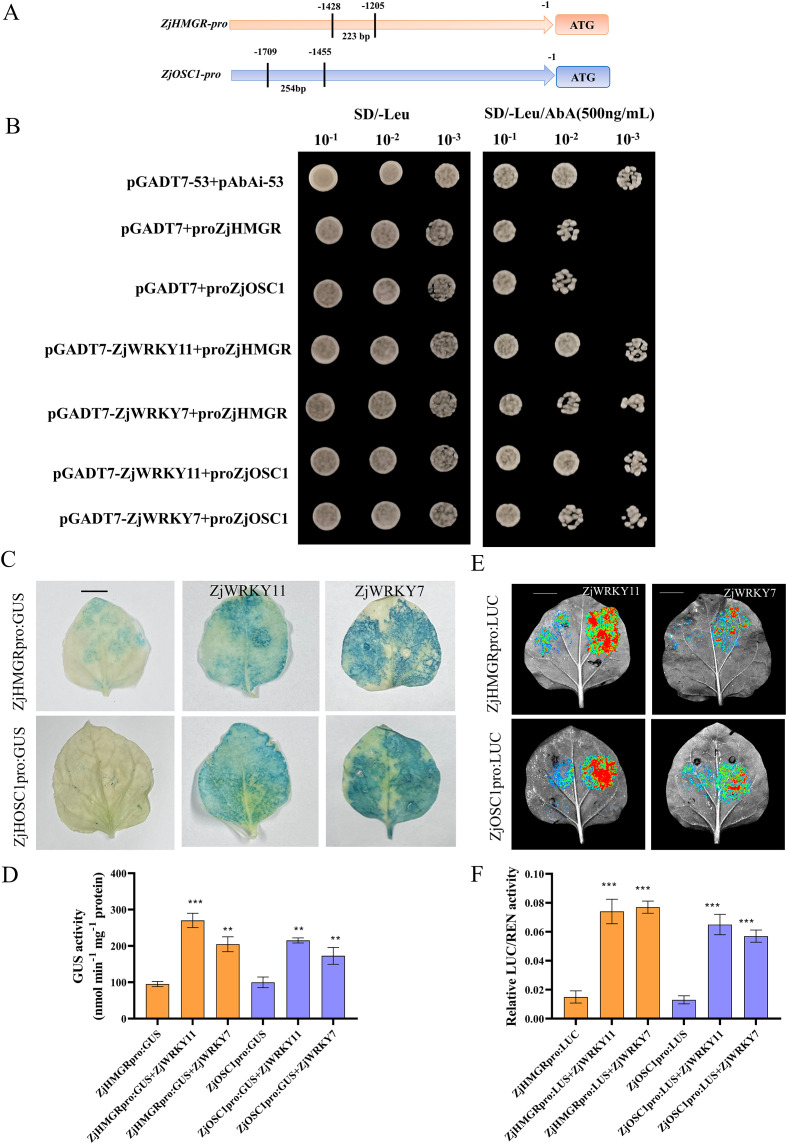
*ZjWRKY11* and *ZjWRKY7* activate the promoter activity of *ZjHMGR* and *ZjOSC1*. **(A)** Analysis of cis-regulatory elements in the promoters of *ZjHMGR* and *ZjOSC1*. **(B)** Identification of the interaction between *ZjWRKY11* and *ZjWRKY7* and the promoters of *ZjHMGR* and *ZjOSC1* with yeast one-hybrid assays. All yeast transformants were cultured and imaged under identical experimental conditions. **(C, D)** Schematic diagram of the detection of GUS reporter gene activity by histochemical staining and fluorescent analysis of GUS protein activity; blue color indicates GUS activity. **(E, F)** Schematic diagram of the firefly luciferase activity of the LUC reporter gene and dual-luciferase activity assays; the pseudocolor bar indicates fluorescence intensity. The experiment was repeated three times. The bars indicate mean ± SE. Values marked with asterisks are significantly different from those of control plants (Student’s t-test; p < 0.05). **p < 0.01, ***p < 0.001.

*ZjWRKY11* and *ZjWRKY7* significantly enhanced the expression of *ZjHMGR* and *ZjOSC1*, suggesting that they may directly regulate these genes during triterpenoid biosynthesis in jujube. Promoter analysis identified putative W-box motifs (TTGACC/T), the canonical binding sites of WRKY transcription factors, within the promoter regions of both *ZjHMGR* and *ZjOSC1* (**Figure 7A**). Yeast one-hybrid (Y1H) assays were performed to examine their direct interactions. The promoters of *ZjHMGR* and *ZjOSC1* fused to the AbAr reporter showed no self-activation under 500 ng/mL AbA. However, co-expression with *ZjWRKY11* or *ZjWRKY7* enabled yeast growth under selective conditions, indicating that both transcription factors directly bind to these promoters (**Figure 7B**). Similar results were consistently observed across independent repeat experiments conducted under identical experimental conditions. The original uncropped images are provided in **Supplementary Figure 6**.

To further validate their regulatory roles, GUS and LUC reporter assays were conducted. Transient expression of *ZjWRKY11* and *ZjWRKY7* markedly enhanced GUS staining intensity and enzymatic activity. Quantitative analysis showed that *ZjWRKY11* increased *ZjHMGR* and *ZjOSC1* promoter activities by approximately 3.0- and 2.5-fold, respectively, whereas *ZjWRKY7* increased them by 1.8- and 2.0-fold (**Figures 7C, D**). Consistently, LUC assays confirmed that both transcription factors activated the promoters of *ZjHMGR* and *ZjOSC1*, with *ZjWRKY11* exhibiting a stronger activation effect (**Figures 7E, F**). Collectively, these results indicate that *ZjWRKY11* and *ZjWRKY7* can bind to and activate the promoters of *ZjHMGR* and *ZjOSC1*, supporting their positive regulatory roles in triterpenoid biosynthesis, with *ZjWRKY11* exhibiting a stronger transcriptional activation effect.

## 4 Discussion

Triterpenoids are a major class of plant secondary metabolites that play essential roles in plant growth, environmental adaptation, and stress resistance, while also contributing to the nutritional and medicinal value of crops (Yao et al., 2020; Erb and Kliebenstein, 2020). These compounds are predominantly synthesized via the mevalonate (MVA) pathway, in which 3-hydroxy-3-methylglutaryl-CoA reductase (HMGR) acts as a key rate-limiting enzyme, and oxidosqualene cyclases (OSCs) catalyze the formation of diverse triterpene skeletons (Sheludko, 2010). Increasing evidence indicates that triterpenoid biosynthesis is tightly regulated by phytohormone signaling networks, including jasmonic acid, salicylic acid, and ethylene, which coordinate metabolic reprogramming in response to environmental stimuli (Wasternack and Strnad, 2018; Yang et al., 2025). MT has recently emerged as an important regulator of plant development, stress responses, and secondary metabolism (Arnao and Hernández-Ruiz, 2014; Dou et al., 2022). In the present study, exogenous MT markedly promoted triterpenoid accumulation in jujube leaves in a concentration- and time-dependent manner, with 100 μM identified as the optimal concentration, whereas a higher level (200 μM) exerted an inhibitory effect, indicating a dose-dependent regulatory pattern. Interestingly, triterpenoid accumulation decreased under the 200 μM MT treatment, indicating that MT-mediated regulation operates within an optimal concentration range. Similar dose-dependent responses have been reported in other plant species, where moderate MT levels promote growth and secondary metabolism, whereas excessive concentrations attenuate these effects (Zhang et al., 2015; Yu et al., 2023). The reduced accumulation observed at 200 μM may be associated with feedback regulation or disruption of hormonal homeostasis, leading to weaker induction of MT-responsive transcription factors and triterpenoid biosynthetic genes. Such a hormetic response likely contributes to maintaining metabolic balance and preventing excessive carbon investment in secondary metabolism. Further studies are required to clarify the regulatory basis underlying the inhibitory effects of high MT concentrations. Notably, MT accumulation following MT treatment preceded the increase in triterpenoid content, suggesting that MT may function as an upstream signaling molecule involved in the activation of downstream transcriptional networks rather than acting as a direct precursor in triterpenoid biosynthesis (Fu et al., 2022; Wei et al., 2015). The delayed accumulation of triterpenoids following MT treatment further supports this interpretation. Although MT levels increased rapidly after treatment, significant changes in triterpenoid content were only detected at later stages, suggesting that MT-mediated regulation involves multiple intermediate processes.

Consistent with this result, transcriptomic analysis revealed extensive transcriptional reprogramming following MT treatment, with differentially expressed genes significantly enriched in secondary metabolic pathways, particularly those associated with terpenoid biosynthesis (Debnath et al., 2020; Xu et al., 2024). This temporal lag likely reflects the sequential activation of signaling cascades, transcriptional regulators, and downstream biosynthetic genes. Our transcriptomic analysis further showed that MVA-pathway genes, particularly *ZjHMGR* and *ZjOSC* were transcriptionally activated by MT, whereas most MEP-pathway genes showed limited responses (**Supplementary Table 9**). This is consistent with the predominant role of the MVA pathway in pentacyclic triterpenoid biosynthesis (Dong and Qi, 2025; Wen et al., 2022) and supports our focus on this pathway, although minor contributions from the MEP branch cannot be completely excluded. In particular, MT-induced expression of *ZjWRKY11* and *ZjWRKY7* may subsequently activate key triterpenoid biosynthetic genes, leading to enzyme accumulation and enhanced metabolic flux toward triterpenoid production. Collectively, these results suggest that MT functions as a signaling cue that activates downstream transcriptional regulatory networks, thereby promoting triterpenoid biosynthesis in jujube. However, because exogenous MT was repeatedly applied, the measured MT content may reflect both absorbed exogenous MT and endogenous MT, which warrants further investigation.

While this study shows that MT induces *ZjWRKY11* and *ZjWRKY7* expression and that both transcription factors positively regulate triterpenoid biosynthesis, whether they are required for the full triterpenoid response to MT remains to be determined. Current evidence suggests that MT perception may involve the putative receptor PMTR1/CAND2 and downstream signaling components, including ROS, Ca^2+^ fluxes, MAPK cascades, and hormone signaling networks (Wei et al., 2018). Because WRKY transcription factors are frequently regulated through these pathways, *ZjWRKY11* and *ZjWRKY7* may function as downstream targets of MT signaling. Future studies combining MT treatment with loss-of-function analyses of *ZjWRKY11* and *ZjWRKY7* will help clarify their precise contribution to MT-induced triterpenoid accumulation and further refine the regulatory model proposed here. Beyond its mechanistic significance, the MT-mediated regulatory pathway identified here also has potential practical value for enhancing triterpenoid production. While exogenous MT treatment effectively increased triterpenoid accumulation, its large-scale application may be constrained by economic and management considerations. In contrast, the MT-responsive transcription factors *ZjWRKY11* and *ZjWRKY7* represent promising targets for molecular breeding and metabolic engineering. Manipulation of these regulators may provide a more sustainable and efficient strategy for improving triterpenoid production in jujube and other medicinal plants.

Transcription factors are key mediators that translate upstream signals into specific metabolic outputs. Among them, WRKY proteins have been widely implicated in the regulation of plant secondary metabolism and stress responses (Li et al., 2025b; Song et al., 2023). Several WRKY transcription factors have been shown to directly regulate terpenoid biosynthesis in other species. For example, *AaWRKY1* activates artemisinin biosynthetic genes in *Artemisia annua* (Han et al., 2014), *SlWRKY73* modulates monoterpene and sesquiterpene accumulation in tomato (Coppola et al., 2019), and *PjWRKY* regulates triterpenoid biosynthesis in *Panax japonicus* (Wang et al., 2026). In this study, integrative analysis of transcriptomic data and gene expression patterns identified several WRKY transcription factors responsive to MT, among which *ZjWRKY11* and *ZjWRKY7* showed strong induction and a high correlation with triterpenoid accumulation. Functional analyses further demonstrated that overexpression of *ZjWRKY11* and *ZjWRKY7* significantly enhanced triterpenoid content, whereas their silencing led to a marked reduction, confirming their positive regulatory roles. In contrast, *ZjWRKY22* did not exhibit a significant effect on triterpenoid levels, highlighting the functional specificity among WRKY family (Chen et al., 2017). These results indicate that *ZjWRKY11* and *ZjWRKY7* are MT-responsive transcription factors that positively regulate triterpenoid biosynthesis and may contribute to MT-associated triterpenoid accumulation. Similar roles of WRKY transcription factors have been reported in other plant species, where they modulate the production of diverse secondary metabolites, including terpenoids, flavonoids, and alkaloids (Zhang et al., 2023b; Huang et al., 2023). Our findings extend this regulatory paradigm to jujube and provide evidence that MT-responsive WRKY transcription factors are involved in controlling triterpenoid accumulation.

**Figure 8:**
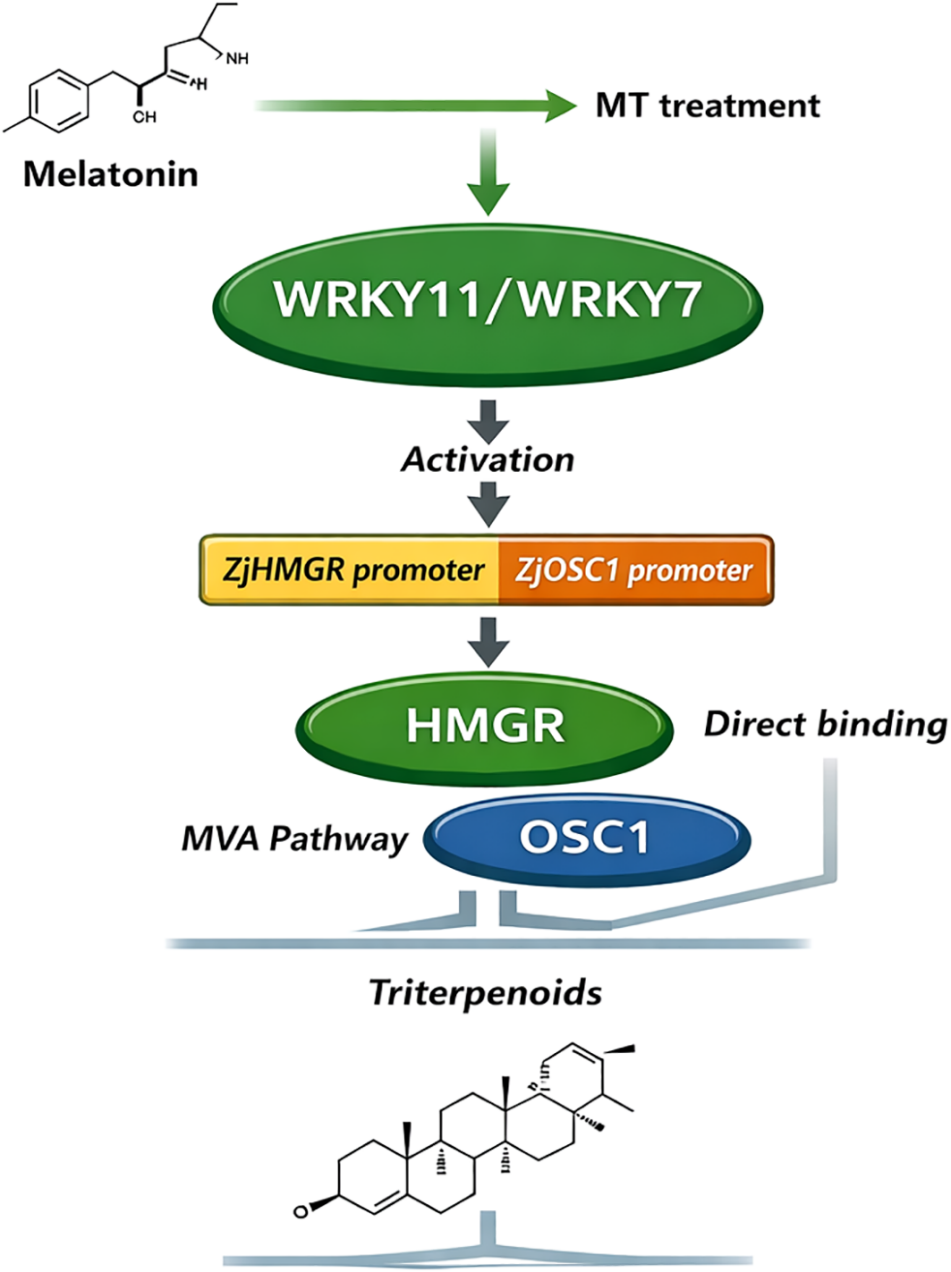
Proposed model illustrating the potential contribution of MT-responsive transcription factors *ZjWRKY11* and *ZjWRKY7* to triterpenoid biosynthesis in jujube.

A major contribution of this study is the elucidation of the direct regulatory mechanism underlying WRKY−mediated triterpenoid biosynthesis. *ZjHMGR* and *ZjOSC1* are two key structural genes in the mevalonate pathway and the triterpene biosynthesis branch. Their functions have been proven to play significant roles, as overexpression of these genes will increase triterpene accumulation, while silencing will have the opposite effect. This is similar to the regulatory functions of *HMGR* and *OSC* genes in other species in controlling the synthesis of terpenoids (Sun et al., 2025; An et al., 2021). Crucially, multiple lines of evidence, including yeast one−hybrid, GUS staining, and luciferase reporter assays, demonstrated that *ZjWRKY11* and *ZjWRKY7* activate the promoters of *ZjHMGR* and *ZjOSC1* and likely enhance metabolic flux toward triterpenoid biosynthesis, providing direct evidence that WRKY transcription factors can transcriptionally regulate key enzymatic genes in the triterpenoid biosynthetic pathway. However, additional downstream biosynthetic and modification enzymes may also contribute to the accumulation of specific triterpenoid compounds. Similar direct regulatory mechanisms have been reported in other plant systems. For example, *AaWRKY1* activates *ADS* and *CYP71AV1* promoters, enhancing artemisinin production in *Artemisia annua* (Ma et al., 2009). *CbWRKY24* enhances saponin production in C. blinii by regulating genes involved in terpenoid biosynthesis (Sun et al., 2018) These examples, together with our findings, highlight that WRKY transcription factors often act as direct activators of biosynthetic genes in terpenoid networks, underscoring a conserved regulatory strategy across diverse plant taxa. Notably, *ZjWRKY11* consistently exhibited stronger transcriptional activation than *ZjWRKY7*, suggesting potential functional divergence between these homologs, which may arise from differences in DNA−binding affinity, promoter context specificity, or transactivation capacity. Functional differentiation among closely related WRKY family members has also been observed in other systems, such as the differential roles of *AtWRKY18/40/60* in stress and metabolic pathways in *Arabidopsis thaliana* (Chen et al., 2010), indicating that such diversification may contribute to fine−tuning of metabolic regulation in response to developmental and environmental cues. Our transcriptome data further showed that *ZjOSC1* was the most prominently MT-responsive OSC family member, whereas other OSC genes exhibited marginal or no significant expression changes under MT treatment (**Supplementary Table 9**). This expression pattern supports the selection of *ZjOSC1* for functional characterization. Based on these findings, we propose a regulatory model in which MT induces the expression of *ZjWRKY11* and *ZjWRKY7*, and these transcription factors positively regulate *ZjHMGR* and *ZjOSC1* expression, thereby contributing to triterpenoid biosynthesis in jujube (**Figure 8**). Although *ZjHMGR* and *ZjOSC1* appear to be important downstream targets of *ZjWRKY11* and *ZjWRKY7*, the accumulation of diverse pentacyclic triterpenoids cannot be fully attributed to the regulation of these two genes alone. These results suggest that MT-responsive triterpenoid biosynthesis is likely associated with the coordinated transcriptional regulation of multiple biosynthetic genes and regulatory components.

In summary, this study elucidates a MT-mediated transcriptional mechanism underlying triterpenoid biosynthesis in jujube. We demonstrate that MT acts as an upstream signal to regulate WRKY transcription factors, which directly activate key biosynthetic genes and thereby promote triterpenoid accumulation. This work highlights a mechanistic link between hormonal signaling and secondary metabolism, expanding our understanding of transcriptional regulation in plant metabolic pathways. Furthermore, these findings provide a conceptual framework for manipulating triterpenoid biosynthesis through targeted regulation of transcription factors. While exogenous MT application may offer a practical approach for enhancing triterpenoid accumulation in high-value crops, the regulatory components identified in this study may facilitate the development of breeding and genetic engineering strategies that achieve similar metabolic outcomes in a more durable and economically sustainable manner. Future studies focusing on upstream signaling networks and downstream metabolic flux will further refine our understanding of MT-regulated secondary metabolism.

## 5 Conclusion

This study provides insights into the transcriptional regulation associated with MT-induced triterpenoid accumulation in jujube. We demonstrate that *ZjWRKY11* and *ZjWRKY7* are MT-responsive transcription factors that positively regulate triterpenoid biosynthesis and may contribute to MT-associated triterpenoid accumulation. These findings deepen our understanding of the transcriptional regulation underlying triterpenoid metabolism and emphasize the pivotal role of transcriptional control in secondary metabolism. Moreover, the regulatory components identified here represent promising targets for metabolic engineering and genetic improvement of jujube and other medicinal plants.

## Data Availability

The datasets presented in this study can be found in online repositories. The names of the repository/repositories and accession number(s) can be found in the article/**Supplementary Material**.
