# Supplementary material for: Melatonin-induced ZjWRKY11 and ZjWRKY7 regulate triterpenoid biosynthesis in jujube (Ziziphus jujuba Mill.)
Source: Front Plant Sci. 2026 Jul 28;17:1868423. doi: 10.3389/fpls.2026.1868423 (PMC13457633; doi:10.3389/fpls.2026.1868423)
Supplement: Supplementary file 1 [file DataSheet1.pdf]

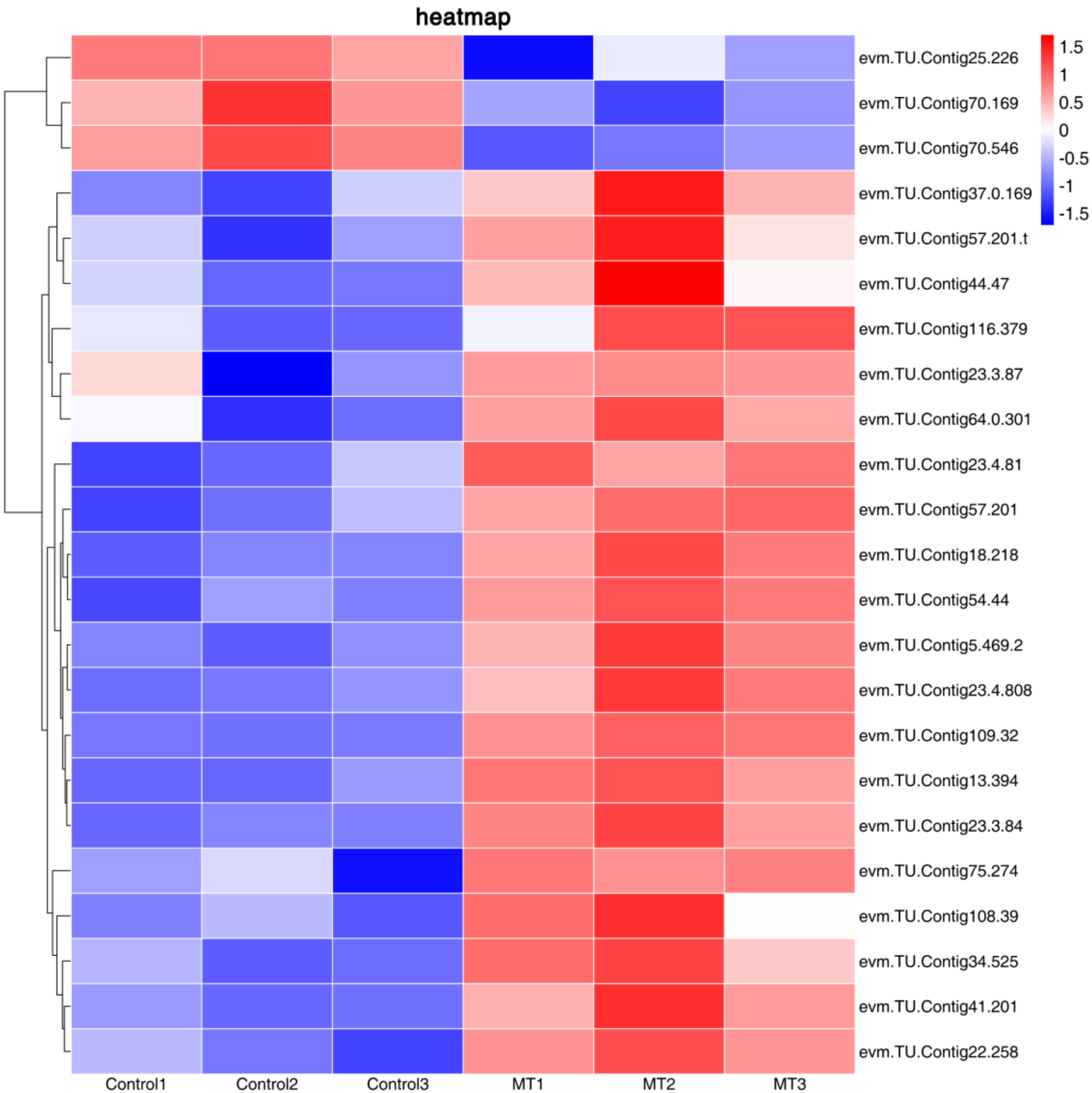

Figure S1. Expression heatmap of all MT-responsive WRKY genes identified from the RNA-seq dataset.

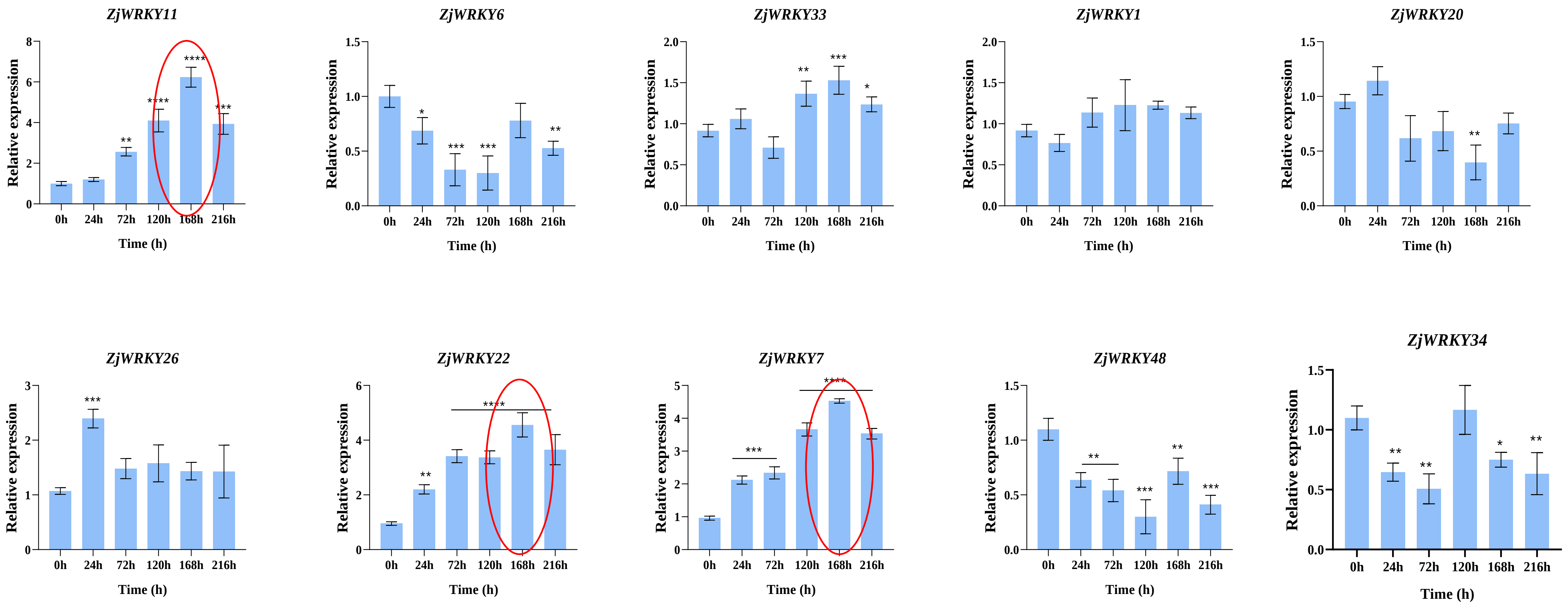

Figure S2. Expression patterns of selected candidate WRKY transcription factors used for further functional analyses.

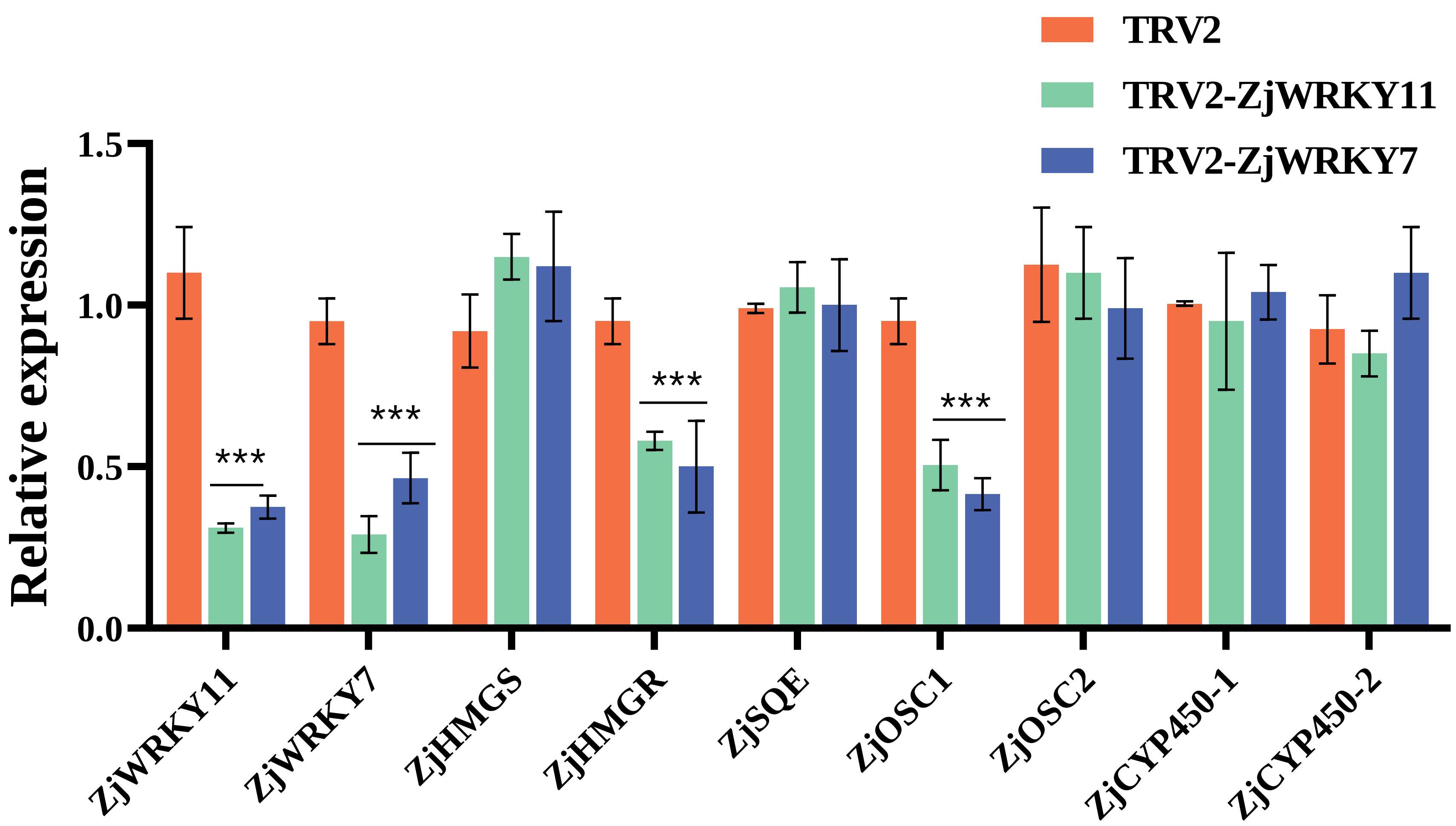

Figure S3. The effects of transiently silent transcription factors ZjWRKY11 and ZjWRKY7 on triterpenoid biosynthesis pathway genes

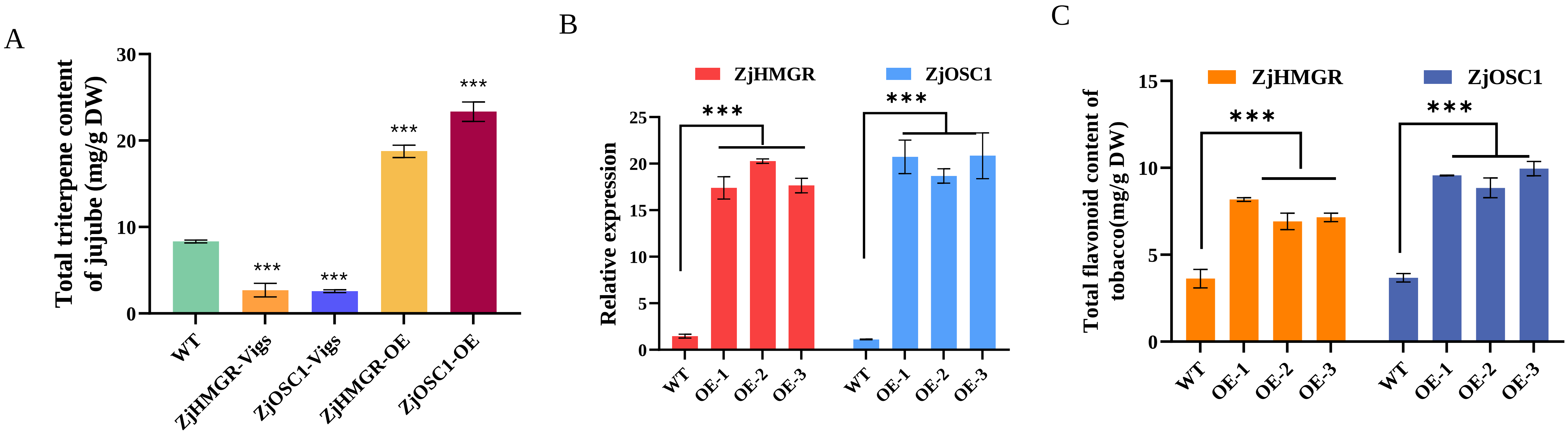

Figure S4. Functional analysis of key genes ZjHMGR and ZjOSC1 for triterpenoid synthesis. (A) Instantaneous transformation to verify the functions of ZjHMGR and ZjOSC1 in triterpenoid synthesis; (B) Measurement of the expression levels of key genes in overexpressing plants; (C) Determination of the total triterpenoid content in overexpressing tobacco plants.

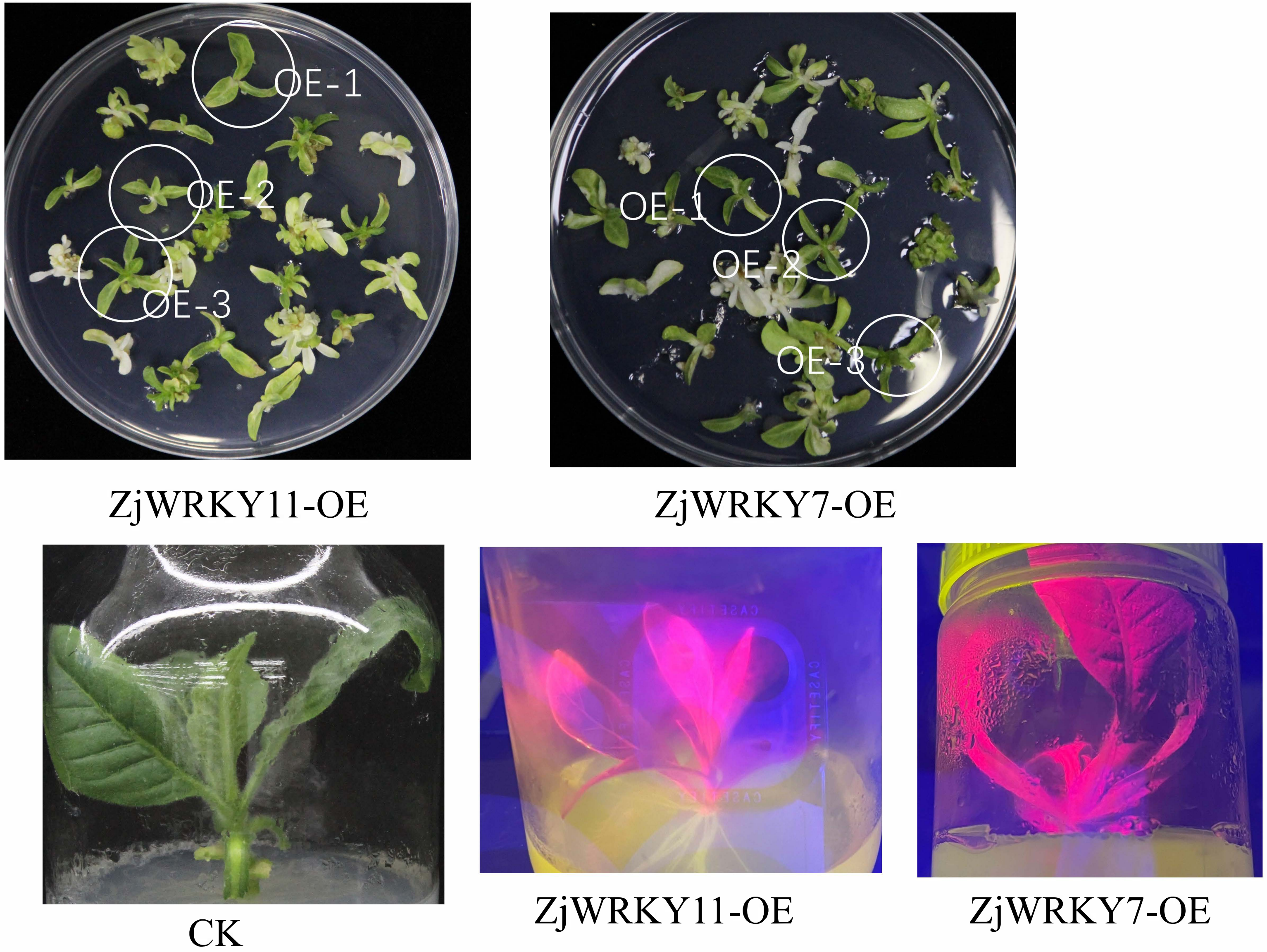

Figure S5. Overexpression of ZjWRKY11/7 in tobacco screening process. Leaves from four-week-old transgenic tobacco plants were used for triterpenoid determination.

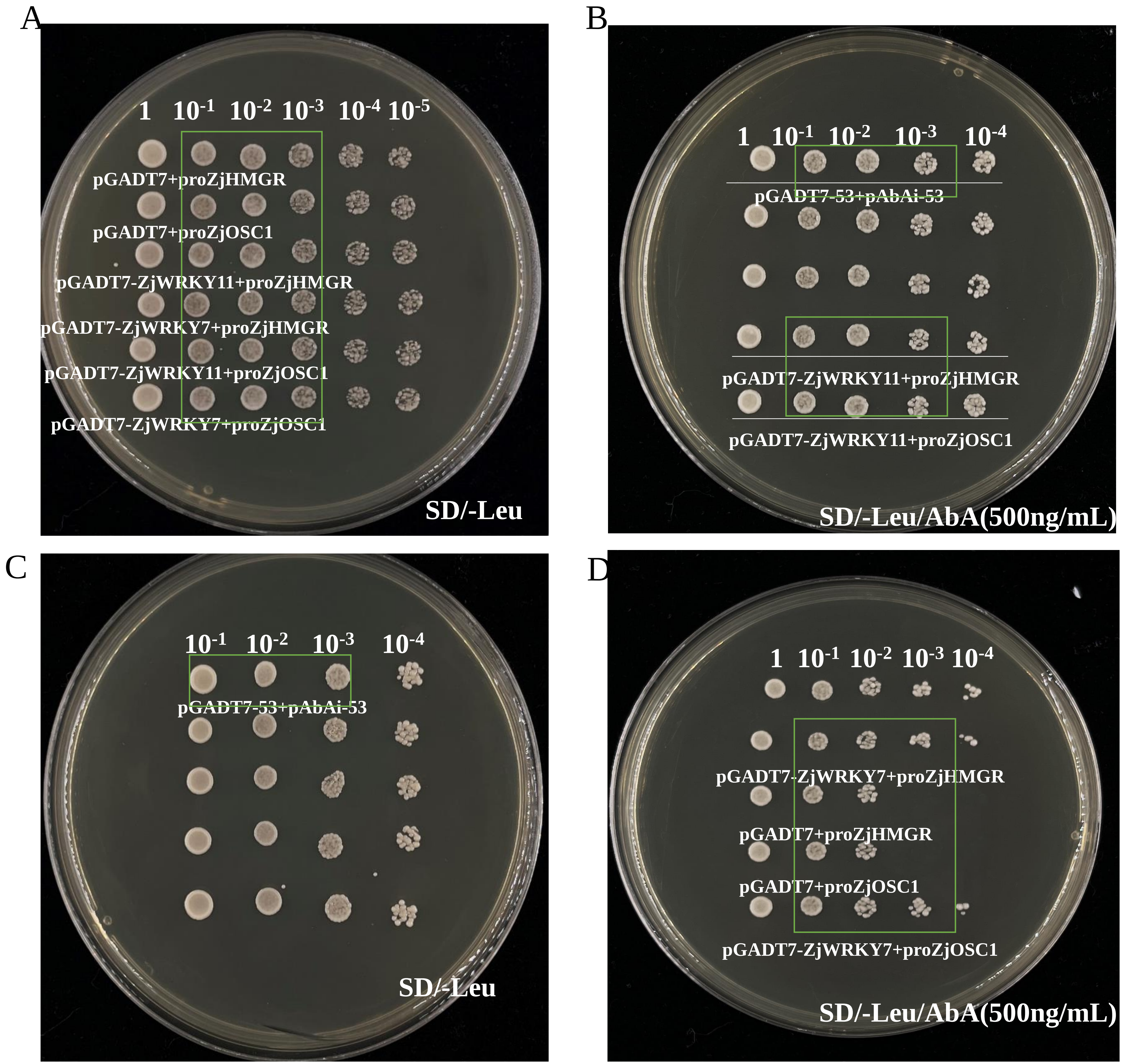

Figure S6. Original uncropped and annotated images of yeast one-hybrid assays corresponding to Figure 7B. (A-D) Original images obtained from independent repeat experiments. All images have been annotated to identify the corresponding yeast transformants. The green box shows the spots used in the result section.
